# 1671. Impact of COVID-19 Pandemic on Rates of Positive Blood Cultures and Contaminations in Children

**DOI:** 10.1093/ofid/ofad500.1504

**Published:** 2023-11-27

**Authors:** Hiroyuki Aiba, Masaki Yamada, Toshihiro Matsui, chikara Ogimi

**Affiliations:** National Center for Child health and development, Setagaya, Tokyo, Japan; National Center for Child health and development, Setagaya, Tokyo, Japan; National Center for Child Health and Development, Setagaya-ku, Tokyo, Japan; National Center for Child Health and Development, Setagaya-ku, Tokyo, Japan

## Abstract

**Background:**

The global COVID-19 pandemic has led to many changes in infection prevention policies and practices in healthcare settings. For example, healthcare providers need to use personal protective equipment during all procedures, including phlebotomy. Blood culture is a widely used test for diagnosing bloodstream infection, and a few publications have reported an increase in its contamination rate in the pandemic era. However, the changes in the culture contamination rate and bacteremia in children have barely been assessed. Hence, we performed a retrospective review of blood culture results during the COVID-19 pandemic era.

**Methods:**

A retrospective, observational study was performed at a tertiary children's hospital in Japan.

The monthly numbers of blood cultures drawn and the blood culture results of the children aged ≤ 18 years who visited the emergency room (ER) at our center from January 2017 to December 2022 were collected using electronic medical records and a clinical database. Of note, all positive culture results were reviewed by pediatric infectious specialists and were interpreted whether the results were true positive (bacteremia) or contaminated. The study period was divided into two eras for comparison: the pre-COVID-19 pandemic era (January 2017-December, 2019) and the pandemic era (January, 2020-December, 2022). Mann-Whitney U test was used for analysis, with a p-value of less than 0.05 being significant.

**Results:**

Overall, the number of pediatric patients presented to the ER was 83,224 in the pre-pandemic period and 57,742 during the pandemic period (Figure 1). The numbers of blood culture specimens were 12,571 and 9,409, respectively. The median (interquartile range) monthly numbers of blood cultures drawn per 1,000 patient visits were 148 (137–164) and 156 (144–173) (p = 0.12) (Figure 2). Similarly, the contamination rates were at 1.5 (0.8–2.1) and 1.7 (1.9–2.3) (p = 0.53), while the rates of patients with bacteremia were 2.1 (1.6–3.0), and 2.9 (2.1–4.3), the latter being significantly higher (p = 0.02).

**Figure:**
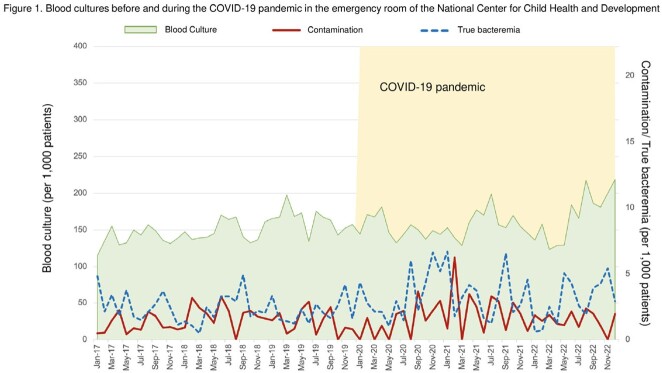

January 2020 was defined as the COVID-19 pandemic.

Comparison by Mann-Whitney U test before and during the COVID-19 pandemic

**Figure:**
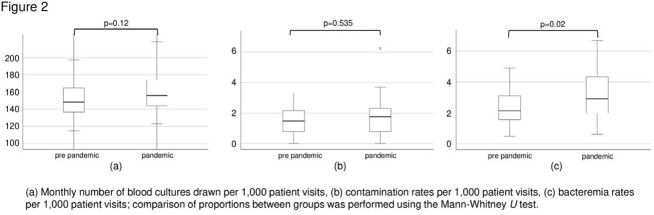

**Conclusion:**

The COVID-19 pandemic did not appear to increase the blood culture contaminants in the pediatric ER at a tertiary center. However, the rate of bacteremia increased during the pandemic. Explanations for this observation will continue to be investigated.

**Disclosures:**

**chikara Ogimi, MD**, bioMerieux Japan Ltd.: Honoraria|Horiba: Honoraria|Pfizer: Honoraria

